# The river runs through it: The Athabasca River delivers mercury to aquatic birds breeding far downstream

**DOI:** 10.1371/journal.pone.0206192

**Published:** 2019-04-09

**Authors:** Craig E. Hebert

**Affiliations:** Environment and Climate Change Canada, Science and Technology Branch, National Wildlife Research Centre, Ottawa, ON, Canada; Brigham Young University, UNITED STATES

## Abstract

This study examined factors contributing to temporal variability (2009–2017) in total mercury (THg) concentrations in aquatic bird eggs collected in the Peace-Athabasca Delta and Lake Athabasca in northern Alberta. Factors examined included year of egg collection, site of collection, bird species, bird diets, annual surface-mineable oil sands production, forest fires, and flow of the Athabasca River. Surface mining activities associated with Alberta’s Athabasca oil sands are situated north of Fort McMurray, Alberta, adjacent to the northward-flowing Athabasca River. Previous studies have found that oil sands industrial operations release mercury into the local (within ~50 km) environment. An information-theoretic approach revealed that the best model for explaining egg THg levels included Athabasca River flow, bird food source, and bird species. Variability in egg THg levels was partly a reflection of differences in food sources, e.g. proportions of aquatic versus terrestrial food in bird diets. Annual fluctuations in maximal flow of the Athabasca River were also important with eggs collected following years of high maximal flow exhibiting higher THg concentrations. Furthermore, eggs collected in years of high versus low flow differed in their stable Hg isotope composition with less mass-independent fraction of ^199^Hg and ^201^Hg in years of high flow. Riverine processes associated with suspended sediment were likely critical in regulating Hg availability to nesting birds. This study highlights the importance of the Athabasca River as a conduit for Hg transport to ecologically-sensitive downstream ecosystems such as the Peace-Athabasca Delta and Wood Buffalo National Park (a UNESCO World Heritage Site). Human activities that increase atmospheric Hg deposition to the Athabasca River watershed, or that enhance Hg releases to the river through erosion of Hg-bearing soils, will likely increase the availability of Hg to organisms inhabiting downstream areas.

## Introduction

**Fig 1:**
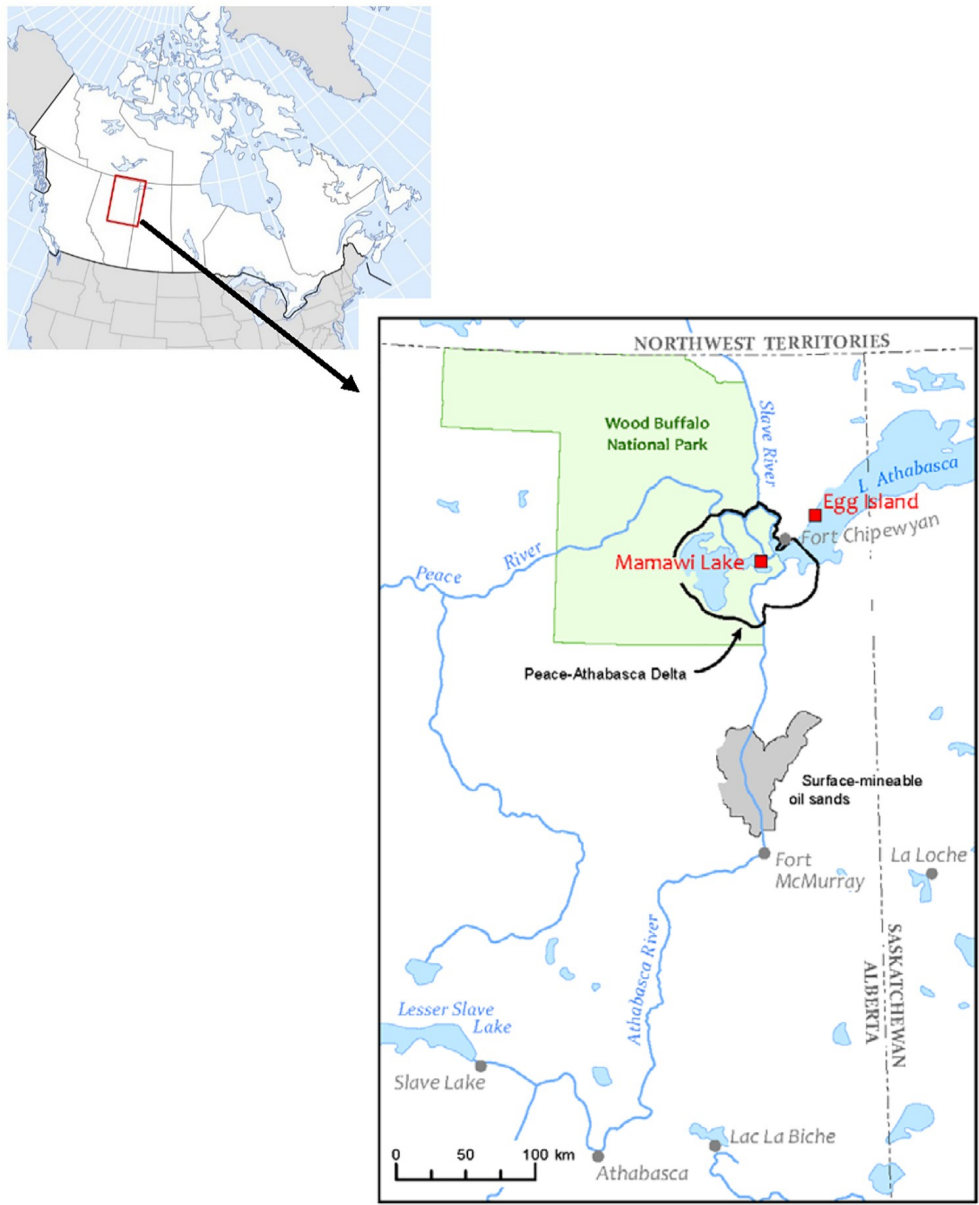
Map of study area showing locations (Mamawi Lake and Egg Island) where aquatic bird eggs were collected in northern Alberta, Canada, 2009–2017.

The Athabasca River is a major river flowing northeast 1200 km from the Rocky Mountains to Lake Athabasca. Along the way, it passes through Alberta’s oil sands, a region of large-scale, open-pit mines used to extract bitumen for synthetic oil production. The river discharges into the Peace-Athabasca Delta (PAD) and western Lake Athabasca approximately 200 km downstream of Fort McMurray (Fig 1).

Since 1967, when oil sands operations commenced, industrial exploitation of the bitumen-rich oil sands has expanded greatly [1]. Previous research has indicated that industrial development associated with the oil sands is a source of mercury (Hg) to the environment [2, 3]. For example, Kirk et al. [3] found that deposition of both total mercury (THg) and methyl mercury (MeHg) in snow resembled a bullseye pattern with higher Hg levels in snow collected closer to oil sands developments. However, snow Hg levels declined rapidly with distance from such developments with most Hg deposited within approximately 50 km. These studies [2, 3] highlighted the possibility that spring snowmelt could result in the release of chemicals, such as Hg, into the aquatic environment. Similarities in relative concentrations of metals in snow and river water provided evidence of metals emitted to the air finding their way into the Athabasca River and tributaries [2]. Kelly et al. [2] also found that Hg concentrations in water were greatest downstream of areas disturbed by oil sands development. Passage of water through the surface-mineable oil sands region, in itself, was not responsible for elevated water Hg levels. Degree of land disturbance caused by oil sands operations was important in enhancing water Hg concentrations.

As oil sands operations are a known source of Hg [2, 3], it is plausible that increases in oil/bitumen production might be accompanied by increases in Hg releases to the environment and uptake into food webs. Oil production from surface mineable sources in the Athabasca oil sands increased greatly through time (S1 Fig). For example, from 2009–2017, oil production from this source increased by 60% (approximately 761,000 bpd (barrels per day) to 1,227,000 bpd) and accounted for approximately 46% of total oil sands production in 2017 (in-situ production accounted for the remainder) [1]. However, potential oil sands Hg contributions need to be evaluated in light of other factors, i.e. organism diet, forest fires, and riverine processes, that may also regulate Hg levels in biota.

Organism diets (particularly as they reflect food source and trophic position) are important in regulating biomagnifying contaminant, e.g. MeHg, concentrations in biota [4–7]. Forest fires could also affect Hg levels in local/regional food webs as they are known to release Hg into the environment [8, 9]. Alberta experienced very large fires in some recent years [10]. Previous research has also highlighted the possibility that the Athabasca River may be a source of Hg to wildlife inhabiting downstream environments [11]. In a large-scale spatial assessment of Hg levels in western Canadian gull eggs, Dolgova et al. [6] found that levels were greatest in eggs collected from breeding sites in receiving waters of the Athabasca River. A critical factor that may regulate Hg transport via the Athabasca River is maximal river flow. Long and Pavelsky [12] documented the influence of river flow on sediment transport down the Athabasca River. Riverine transport of sediment-bound Hg is an important mechanism for Hg transfer to downstream environments [13, 14]. Suspended sediment concentrations (SSC) may also influence Hg dynamics in the environment through effects on the photochemical reduction of MeHg. These effects, and possible Hg sources, can be evaluated using Hg stable isotopes. Mass-dependent and mass-independent fractionation of stable Hg isotopes can provide insights into processes regulating Hg dynamics in the environment.

**Fig 2:**
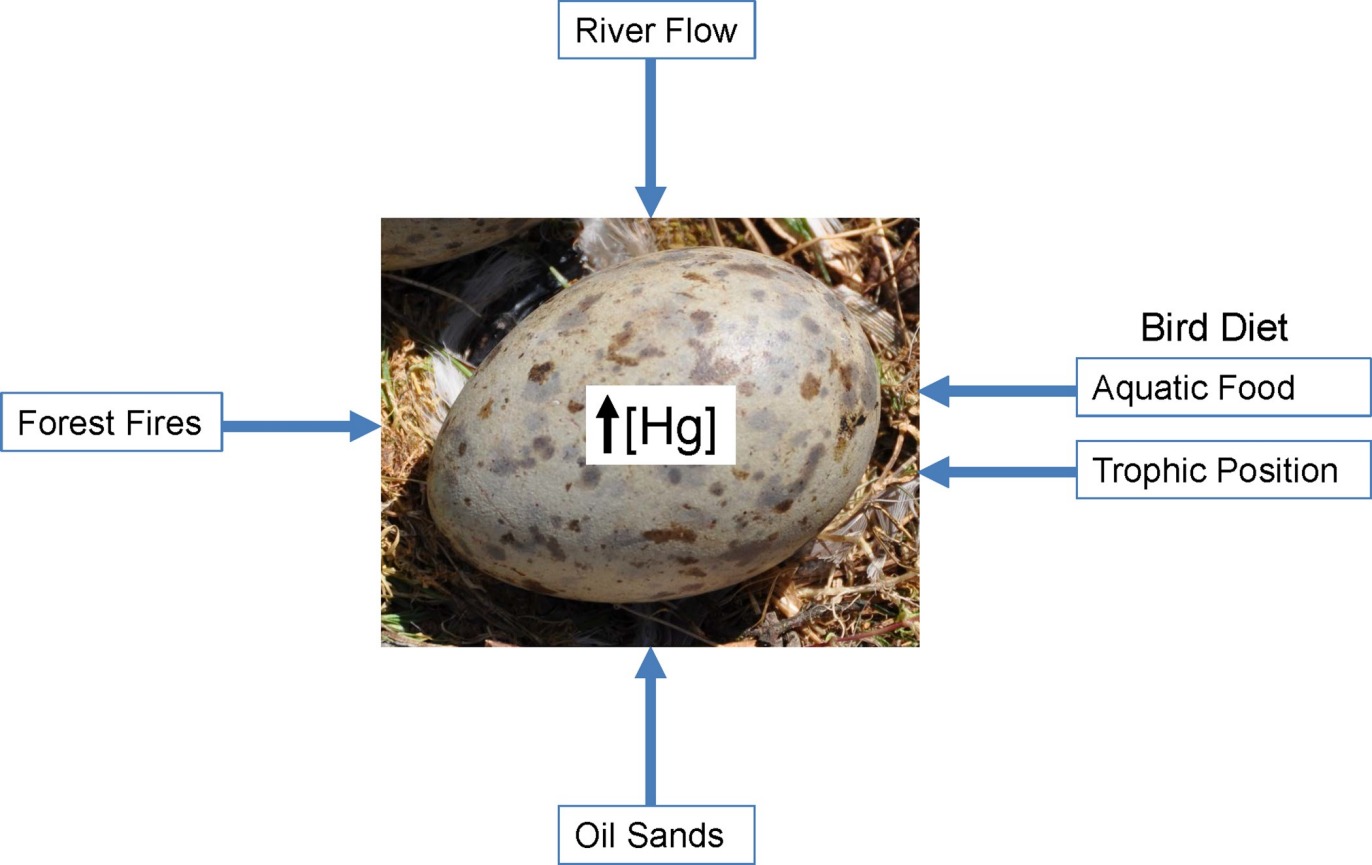
Potential factors influencing Hg concentrations in gull and tern eggs. Increases in any of these factors could increase Hg levels in eggs.

Colonial aquatic birds, e.g. gulls and terns, to monitor the state of the environment has been demonstrated [15]. These birds are top predators resulting in their accumulation of high levels of biomagnifying contaminants. Eggs are a useful sampling matrix for contaminant studies as their collection has little impact on bird populations and eggs of these species are formed from exogenous, locally-obtained resources [16]. Since 2009, gull and tern eggs have been collected annually from sites in WBNP (Mamawi Lake) and western Lake Athabasca (Egg Island). The goal of this study was to examine temporal trends (2009–2017) in egg THg levels and determine how the factors discussed above may be contributing to those trends (Fig 2).

## Methods

### Field methods

Alberta Environment and Parks (18–527), Parks Canada Agency (WB-2018-27577), and the Canadian Wildlife Service (16-AB/SK-SC003) provided the necessary research and collection permits. Animal handling procedures were approved (CH04-2016) under the authority of the National Wildlife Research Centre Animal Care Committee. Gull and tern eggs were collected in June 2009–2017 (in 2010, no collections were attempted) at two sites located in receiving waters of the Athabasca River. Egg Island (59.98N, -110.44W) is located in the western end of Lake Athabasca approximately 40 km northeast of the mouth of the Athabasca River (Fig 1). Mamawi Lake (58.60N, -111.47W) is located in the PAD within the boundaries of Wood Buffalo National Park (WBNP). The PAD is a wetland of international significance [17] and is a defining feature of WBNP, a UNESCO World Heritage Site. It provides habitat for millions of birds and WBNP is the only breeding area for the endangered Whooping Crane (*Grus americana*) [18]. The surrounding region also provides important wildlife habitat. For example, Egg Island is a provincial ecological reserve harboring a variety of colonially-nesting aquatic birds including the largest breeding colony of Caspian Terns (*Hydroprogne caspia*) in Alberta [19]. The PAD and Lake Athabasca are also important to Indigenous land users who rely on traditional wild foods [20].

In most years, 10 eggs were collected per species per site. At Mamawi Lake, Ring-billed Gull (*Larus delawarensis*) and Common Tern (*Sterna hirundeo*) eggs were collected. Low water levels in 2011 prevented access to that location so no eggs were collected that year. Also, in 2014 it was impossible to collect Common Tern eggs at that site. At Egg Island, 10 eggs were collected annually from three species: California Gull (*Larus californicus*), Caspian Tern, and Common Tern. The first recorded nesting of Common Terns on Egg Island was in 2011 so eggs of that species were not collected from that site in 2009. After collection, eggs were immediately transported to the National Wildlife Research Centre (NWRC), Ottawa, ON, Canada in padded cases.

Epiphytic lichen was collected from the branches of coniferous tree species in 2013 (May or July) from 13 locations north, south, east and west of Fort McMurray. All sites were within 100 km of that city. Samples (n = 26, 1–4 samples per site) were cleaned by removing foreign debris, freeze-dried, and pulverized into a fine powder before analysis. The utility of tree lichens as passive collectors of atmospheric pollutants, including mercury, has been demonstrated [21, 22]. Mercury isotope measurements in lichens (see below under Laboratory Methods) were used to establish baseline Hg isotopic “signatures” associated with atmospherically deposited mercury in the Fort McMurray region. This would include mercury from all sources to that region.

### Laboratory methods

Upon arrival at NWRC, eggs were opened and egg contents (i.e. yolk, albumen, embryonic tissue) were homogenized/pulverized using liquid nitrogen and a cryogenic ball-mill. Homogenates were aliquoted into acid-washed glass and polypropylene containers and stored at -40^o^ C for a maximum of three months prior to analysis.

Details regarding egg THg analysis are identical to those reported in Hebert and Popp [7]. Briefly, 20 mg of dried egg contents in nickel boats were delivered by autosampler to a direct Hg analyzer (DMA-80; Milestone). In eggs, approximately 97% of THg is in the organic MeHg form [23] so measuring THg is a cost-effective way to assess MeHg levels in eggs. THg concentrations measured in all samples were within concentration ranges of certified reference materials (OT1566b, TORT-3, DOLT-4, IAEA-407, BCR-463). Limit of detection for THg was 0.006 μg/g (dry weight). In total, 369 eggs were analyzed from a total of 36 species/year/site combinations (S1 Table).

Stable isotopes of nitrogen (^15^N and ^14^N expressed as δ^15^N) and carbon (^13^C and ^12^C expressed as δ^13^C) were measured in the contents of individual eggs to assess bird diets across years. Details regarding stable isotope analysis are identical to those reported in Hebert and Popp [7]. Briefly, stable N and C isotope analyses were conducted using an Elementar Isotope Cube elemental analyzer, followed by trap-and-purge separation and online analysis by continuous flow with a DeltaPlus Advantage isotope ratio mass spectrometer (Thermo Scientific) coupled with a ConFlo III. Dietary change affecting the trophic position of laying females is reflected in egg δ^15^N values (‰), higher trophic position results in greater δ^15^N values. Because Hg biomagnifies, temporal changes in trophic position would be expected to affect female exposure to, and uptake of, MeHg with concomitant effects on egg THg levels. Carbon isotopes are useful in evaluating food sources utilized by consumers [24] and are important for understanding MeHg accumulation in biota as food web utilization, e.g. aquatic versus terrestrial, will influence Hg exposure in consumers [5]. Because lipid content of samples can affect δ^13^C values, a mathematical approach was used to adjust δ^13^C values in eggs based upon C:N ratios in individual samples (C:N ratios for all samples were > 4.0). The equation used to adjust egg δ^13^C values for lipid was from Elliott et al. [25], *δ*^13^C_lipid–corrected_ = *δ*^13^C_non–corrected_—4.46 + 7.32 * Log (C:N ratio). In total, 369 eggs were analyzed from a total of 36 species/year/site combinations (S1 Table).

Stable Hg isotope (^198^Hg, ^199^Hg, ^200^Hg, ^201^Hg, ^202^Hg) analysis was conducted at Trent University’s Water Quality Centre. Details regarding mercury isotope analysis have been reported previously [26]. All Hg isotopes undergo mass dependent fractionation (MDF, reported in delta (δ) notation). MDF of Hg isotopes is influenced by a variety of factors including physical processes as well as by biological reactions [27] and can be useful in identifying the flow of mercury from terrestrial and aquatic systems to consumers [28]. Mass-independent fractionation (MIF) of Hg isotopes is unrelated to isotope mass and is reported in capital delta (Δ) notation. MIF describes the difference between measured δ^xxx^Hg values and scaled δ^202^Hg values (Δ^xxx^Hg = δ ^xxx^Hg − (δ^202^Hg × β)). β, the scaling factor, is determined by theoretical laws of mass dependent fractionation (MDF) and is an isotope-specific constant. MIF has largely been documented for the odd mass Hg isotopes (^199^Hg, ^201^Hg) and is thought to primarily be the result of a magnetic isotope effect, i.e. dissimilarities in magnetic spin of even and odd mass isotopes result in their reacting at different rates. Existing evidence suggests that MIF is not influenced by food web/trophic interactions [29] but occurs during photochemical MeHg degradation and photoreduction of Hg^2+^ [30]. Therefore, factors that affect Hg exposure to light, e.g. light penetration through the water column, are expected to influence MIF of Hg isotopes [31]. Photodegradation of MeHg to Hg^0^ may be an important MeHg removal mechanism, particularly in systems characterized by clear water with a high degree of light penetration, and this can be assessed using Hg isotopes [32]. However, only a portion of the MeHg in such systems will undergo photochemical demethylation. The remaining MeHg will exhibit MIF of ^199^Hg and ^201^Hg and that MeHg may be incorporated into food webs with Hg isotope values in consumers (e.g. birds) reflecting MIF of Hg isotopes [32].

The slope of the MIF of ^201^Hg versus ^199^Hg is useful in differentiating the Hg species (MeHg or Hg^2+^) undergoing photoreduction (MeHg slope ~ 1.3, Hg^2+^ slope ~1.0) [30, 32, 33]. A slope near 1.3 indicates that photoreduction of MeHg is the main mechanism underlying MIF values. If photochemical reduction of MeHg is the main process underlying MIF of Hg isotopes then Δ^199^Hg and/or Δ^201^Hg values in consumer tissues can be used to estimate the amount of MeHg that has been photodegraded [30, 34].

Here, the focus is on the interpretation of MDF (δ^202^Hg) and MIF results (Δ^199^Hg and Δ^201^Hg). Hg isotope analyses were conducted on lichen samples (13 sites) and on eggs from Caspian Terns, Common Terns, California Gulls, and Ring-billed Gull from Egg Island and Mamawi Lake. In total, 256 eggs were analyzed from a total of 26 species/year/site combinations (S1 Table).

### Analytical and statistical methods

Annual estimates (2009–2017) of synthetic oil/bitumen production from surface-mined oil sands deposits were obtained from the Canadian Association of Petroleum Producers (CAPP [1]).

Annual estimates of total forest fire extent (ha) for the province of Alberta were obtained from the National Forestry Database [10]. During the period of study (2009–2017), 99% of total annual area burned occurred during May-August with June-August usually being the focal months. Hence, these fires would have occurred after the egg-laying period in that same calendar year. Caldwell et al. [35] reported sediment MeHg levels peaked within three months after a wildfire, further delays would be expected in terms of wildfire-generated Hg being incorporated into food webs. Therefore, fire extent in the year preceding egg collection was used to assess the impact of fire on egg THg levels. The one-year time lag between forest fire extent and egg THg levels allowed time for Hg released from fires to be incorporated into, and passed through, the food webs utilized by birds.

Athabasca River flow data were obtained from a hydrometric station (07DA001) five km north of Fort McMurray (https://wateroffice.ec.gc.ca/mainmenu/historical_data_index_e.html). June flow was used to estimate maximal annual river flow. Based upon Long and Pavelsky [12], flow thresholds exceeding 1500–1700 m^3^/s resulted in enhanced transport of suspended sediments down the river and into Lake Athabasca. Here, years were categorized as being low (June mean flow <1600 m^3^/s) or high (June mean flow ≥1600 m^3^/s) flow years. River flow in the year preceding egg collections was used to categorize egg collection years according to flow. The one-year time lag between river flow and egg THg levels allowed time for Hg to be incorporated into food webs utilized by terns and gulls [36].

True color Landsat images [37] of the PAD and western Lake Athabasca were obtained from years of low (2002, 2010, 2015) and high (2011, 2014, 2017) Athabasca River flow. These images were used to qualitatively visualize annual differences in the extent of sediment plumes entering western Lake Athabasca.

Data regarding SSC and secchi depth (a measure of water clarity) were obtained from Long and Pavelski [38]. Data were available from sampling sites situated along a southeast-northwest transect crossing the western basin of the lake (see [12]). Distance of each sampling site from the mouth of the Athabasca River was estimated based upon georeferenced data. Data from low (2010) and high (2011) flow years were used to assess inter-year differences in the influence of the river on SSC in western Lake Athabasca and resultant impacts on water transparency.

Comparisons across species and years for egg stable isotope data and for inter-year differences in egg THg levels were assessed using ANOVA or Kruskal-Wallis tests followed by selected post-hoc tests (Tukey’s HSD or Dunn’s test). Correlations between variables were evaluated using Pearson correlation coefficients (*r*).

An information-theoretic approach [39] was used to assess how well candidate models explained annual (2009–2017) variation in THg concentrations in aquatic bird eggs. Models described egg THg concentrations with year of collection, site of collection, bird species, bird food source (δ^13^C), bird trophic position (δ^15^N), oil sands production, annual extent of Alberta forest fires, and Athabasca River June flow as predictor variables. Variables measured on individual eggs were averaged by species and year to facilitate statistical comparison with other predictor data (S2 Table). Differences in Akaike's Information Criterion (AIC) (adjusted for small sample sizes, ΔAICc) were computed for each of 255 models (see S3 Table).

The effects of river flow on species-specific egg THg concentrations and egg Hg isotope values were examined using linear mixed models (LMM) with flow category (high ≥ 1600 m^3^/s; low < 1600 m^3^/s) as a fixed effect and year as a random effect. Year was not significant in any of the models except one (egg THg in Egg Island Common Terns) hence it was removed from subsequent analyses. All statistical analyses were conducted using Statistica Ver 12 [40] with α = 0.05. Student’s two-tailed t-tests were used to compare egg THg levels and Hg isotope values between years of low and high Athabasca River flow. Assumptions underlying the use of parametric statistics were tested using Q-Q plots, the Shapiro-Wilk W test (normality), and Levene’s test (homogeneity of variances).

## Results

### Temporal trends in egg THg concentrations (2009–2017)

**Fig 3:**
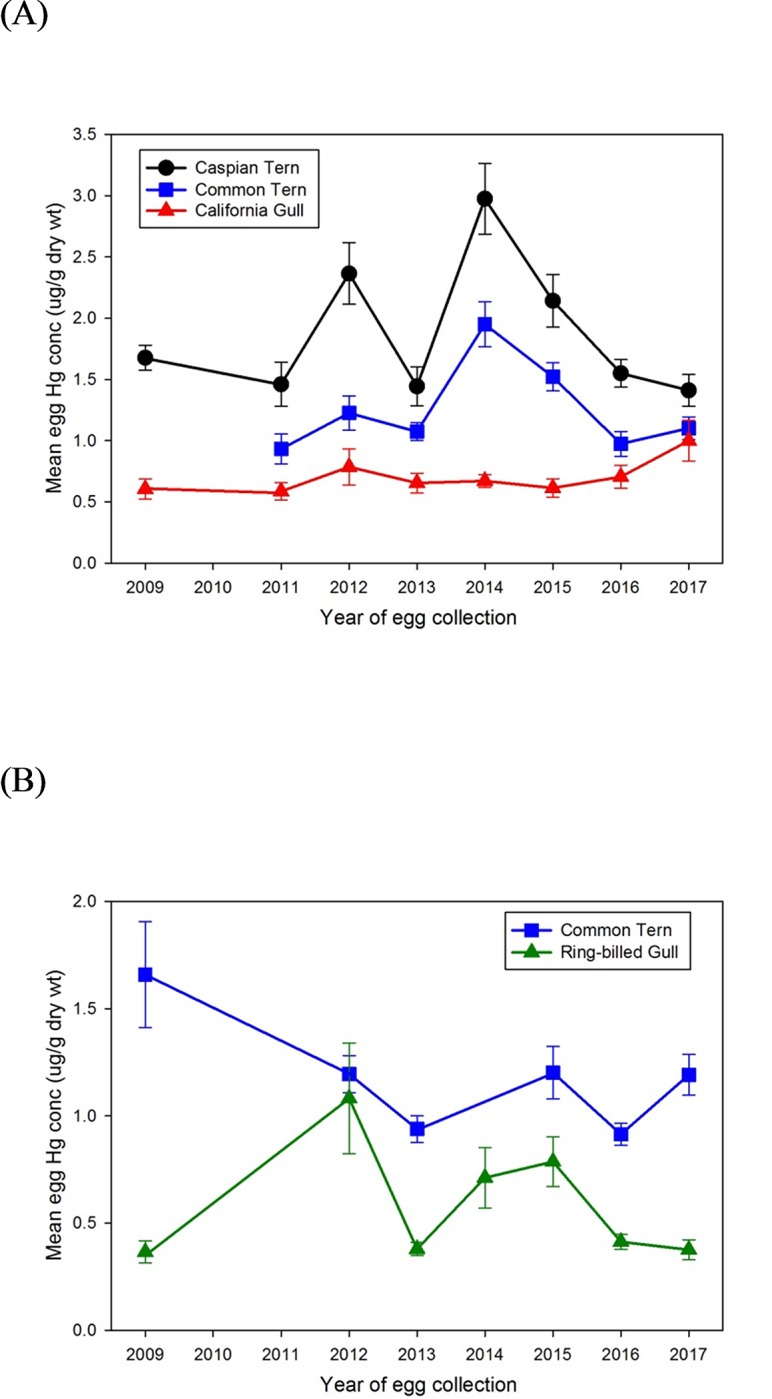
Annual mean (± SE) THg levels (ug/g, dry weight) in gull and tern eggs from sites in receiving waters of the Athabasca River. (a) Egg Island, western Lake Athabasca: Caspian Terns, Common Terns, California Gulls (b) Mamawi Lake: Common Terns, Ring-billed Gulls.

Egg THg concentrations showed inter-year differences for most species/site combinations (Egg Island: Caspian Terns ANOVA *F*(7,72) = 8.83, *p* < 0.001; Common Terns ANOVA F(6,63) = 8.96, *p* < 0.001; Mamawi Lake: Ring-billed Gulls Welch’s ANOVA *F*(6,30) = 3.61, p = 0.01; Common Terns Welch’s ANOVA *F*(5,20) = 3.92, *p* = 0.01) except for Egg Island California Gulls (Welch’s ANOVA *F*(7,31) = 0.89, *p* = 0.53). However, egg THg concentrations were not correlated with year of collection for Egg Island Caspian Terns (n = 80, *r* = -0.01, *p* = 0.97), Egg Island Common Terns (n = 70, *r* = 0.08, *p* = 0.53), or Mamawi Lake Ring-billed Gulls (n = 83, *r* = -0.13, *p* = 0.24) (Fig 3*A* and 3*B*). THg levels in eggs of Egg Island California Gulls increased through time (n = 81, *r* = 0.24, *p* = 0.03) while THg levels in Common Tern eggs from Mamawi Lake decreased (n = 55, *r* = -0.40, *p* = 0.002) (Fig 3*A and* 3*B*).

An information-theoretic approach based upon ΔAICc indicated that egg THg data were best explained by a model including bird species, egg δ^13^C values, and Athabasca River flow (see S3 Table for a complete list of model ΔAICc and weights (w_*i*_^*c*^)).

### Species-specific factors influencing egg THg concentrations

The importance of bird diet, particularly food source, in regulating egg THg levels was partially reflected in inter-specific differences in egg δ^13^C values (S4 Table) (Kruskal-Wallis H(3,369) = 126.46, p < 0.001, Dunn’s test). Egg δ^13^C values were less negative in California Gulls (mean = -23.7‰) than the other three species (mean values; Common Tern = -26.3‰, Caspian Tern = -25.8‰, Ring-billed Gull = -25.7‰). δ^13^C values in Ring-billed Gulls were also less negative than Common Terns. For four of the five species/site combinations, inter-year differences in δ^13^C values were observed but these were minimal with most years having similar values (S4 Table). Inter-specific differences in diets were further reflected in egg δ^15^N values (S5 Table).

**Fig 4:**
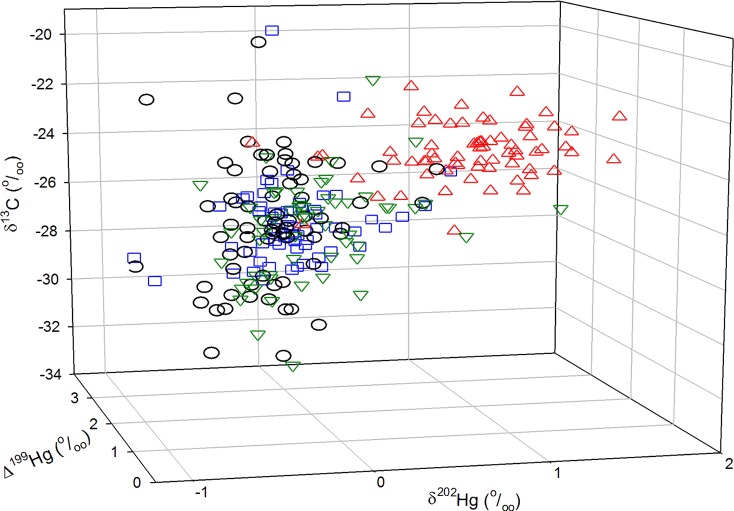
Three-dimensional plot showing lipid-corrected δ^13^C values in eggs as well as mass-dependent fractionation (MDF, as δ^202^Hg) and mass independent fractionation (MIF, as Δ^199^Hg) of Hg in gull and tern eggs. Eggs were collected from Egg Island and Mamawi Lake in 2009–2017. Symbols: circles are Caspian Terns, squares are Common Terns, triangles are California Gulls, inverted triangles are Ring-billed Gulls.

Interspecific comparisons of stable carbon isotope and MDF of stable Hg isotopes (as inferred from δ^202^Hg values) revealed significant differences among species (see Fig 4). Egg δ^202^Hg values were greater in California Gulls than the other bird species (ANOVA *F*(3,252) = 137.6, *p* < 0.001, followed by Tukey’s HSD test). Caspian Terns had lower δ^202^Hg values than either Common Terns or Ring-billed Gulls.

### Influence of riverine processes on egg THg, SSC, water clarity and MIF of Hg isotopes

**Fig 5:**
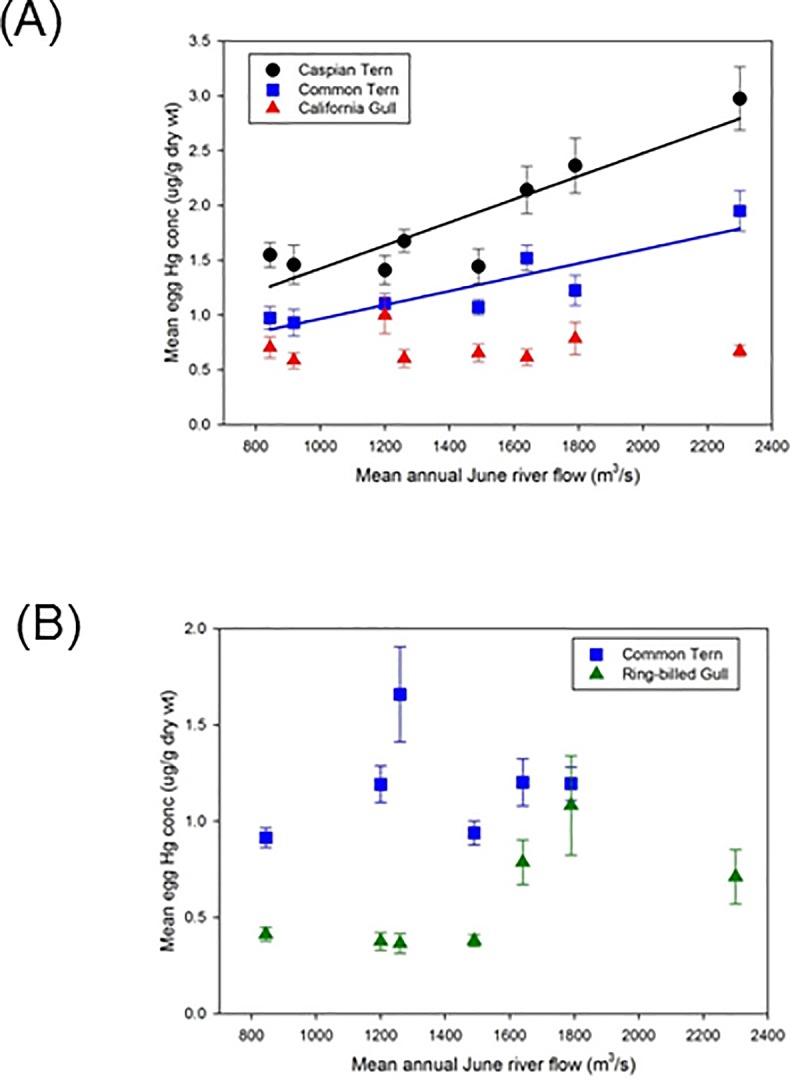
Annual mean June flow (m^3^/s) of the Athabasca River versus annual mean (± SE) THg levels (ug/g dry weight) in gull and tern eggs collected the following year. (a) Egg Island, western Lake Athabasca: Caspian Terns, Common Terns, California Gulls (b) Mamawi Lake: Common Terns, Ring-billed Gulls. Egg THg levels were compared to river flow in the year preceding egg collections.

Annual maximal June flow of the Athabasca River showed large inter-year differences with flow varying approximately three-fold during the period of study (S2 Fig). The Athabasca River is unregulated by dams and annual flow is related to the volume of snowpack and glacier melt [41–43]. Mean annual THg concentrations in eggs were linearly correlated with river flow from the previous year in two of the five species/site comparisons (Egg Island: Caspian Terns n = 8, *r* = 0.90, *p* = 0.003; Common Terns n = 7, *r* = 0.89 *p* = 0.007) (Fig 5).

**Fig 6:**
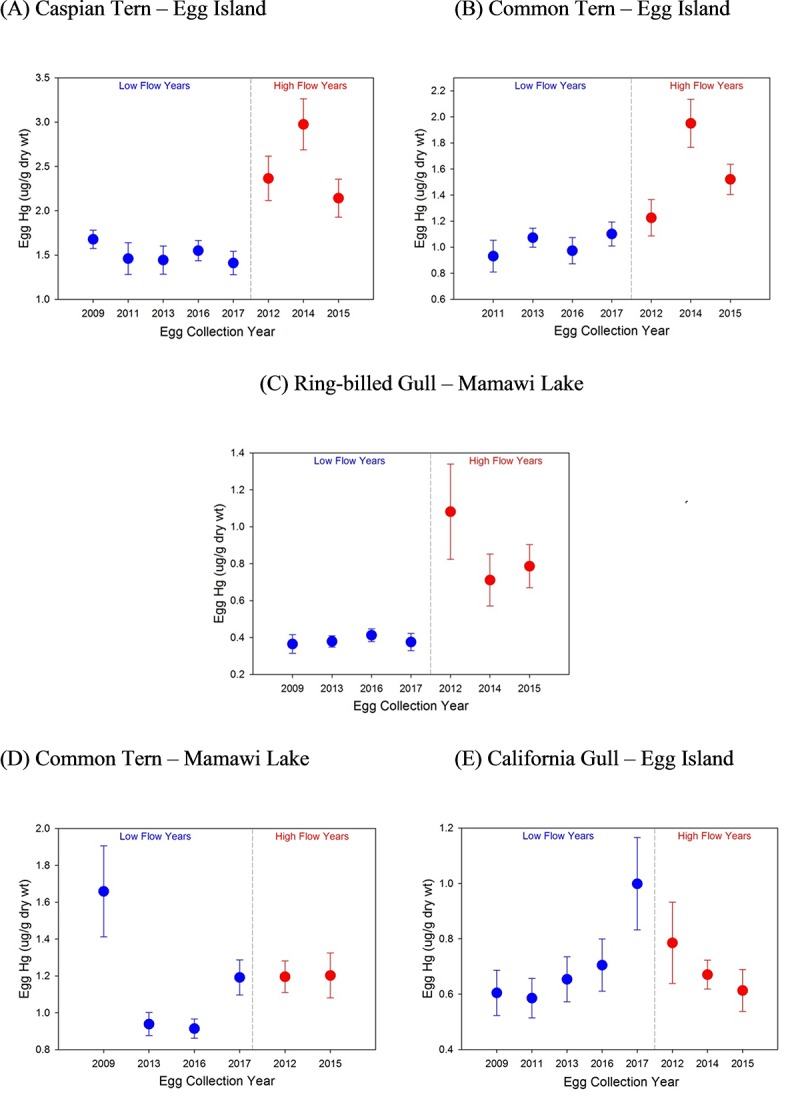
Annual mean (± SE) THg levels (ug/g dry weight) in eggs collected following years of low and high flow in the Athabasca River. Low flow years were categorized as those having a maximal annual flow of less than 1600 m^3^/s. (a) Egg Island Caspian Terns (b) Egg Island Common Terns (c) Mamawi Lake Ring-billed Gulls (d) Mamawi Lake Common Terns (e) Egg Island California Gulls. Egg THg levels were compared to river flow in the year preceding egg collections.

However, visual examination of the data indicated that the relationship between egg THg concentrations and river flow might not be best described by a linear relationship. Instead, a threshold effect seemed more appropriate with greater egg THg levels being observed in years when river flow surpassed a mean June threshold of 1600 m^3^/s. To investigate this further, years were categorized as being low (<1600 m^3^/s) or high (≥1600 m^3^/s) flow based on June mean monthly flow. Years classified as low flow years were: 2008, 2010, 2012, 2015, 2016; high flow years were 2011, 2013, 2014. THg levels in eggs were elevated following high river flow years in the majority of species/sites examined (Fig 6). THg concentrations in eggs collected following high flow years were greater in Caspian Terns (Egg Island) (mean_low_ = 1.51 μg·g^-1^ dry wt, mean_high_ = 2.49 μg·g^-1^ dry wt; *t* = 6.85, 78 *d*.*f*., *p* < 0.0001), Common Terns (Egg Island) (mean_low_ = 1.02 μg·g^-1^ dry wt, mean_high_ = 1.58 μg·g^-1^ dry wt; LMM F(1,5) = 9.76, *p* = 0.03), and Ring-billed Gulls (Mamawi Lake) (mean_low_ = 0.38 μg·g^-1^ dry wt, mean_high_ = 0.86 μg·g^-1^ dry wt; *t* = 5.60, 81 *d*.*f*., *p* < 0.0001). THg concentrations in eggs from these species/sites were on average 82% greater in high flow versus low flow years. Eggs from Egg Island California Gulls (*t* = 0.24, 79 *d*.*f*., *p* = 0.81) and Mamawi Lake Common Terns (*t* = 0.19, 53 *d*.*f*., *p* = 0.85) did not show a significant difference in THg levels between low and high flow years.

**Fig 7:**
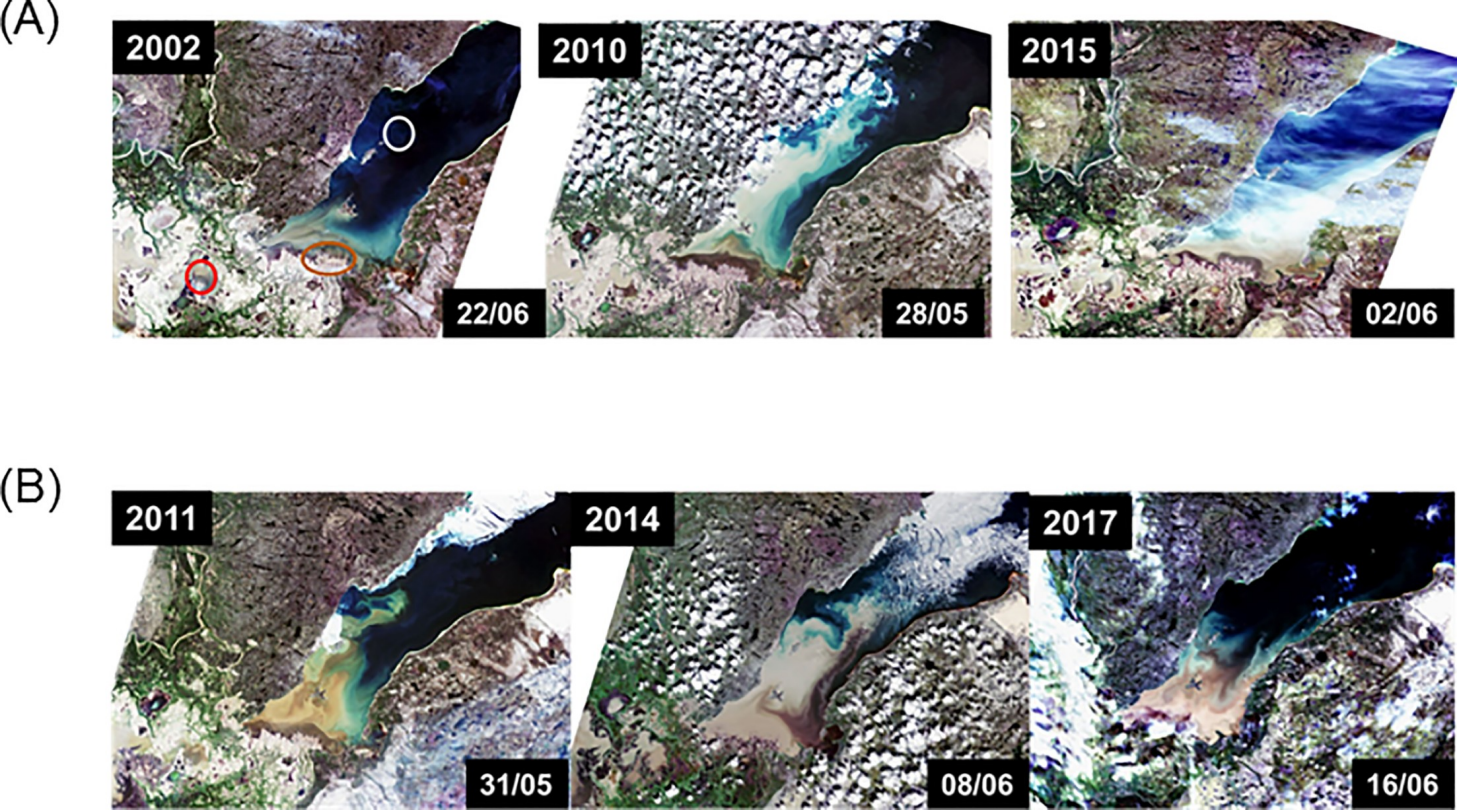
True color Landsat images of the PAD and western Lake Athabasca during years of low (<1600 m^3^/s June flow) and high (≥1600 m^3^/s June flow) Athabasca River flow. In high flow years, there is a notable increase in the extent of the brown sediment plume entering the lake. 2015 image is obscured to some extent by white cloud cover but sediment is clearly visible. Red and white circles indicate the locations of Mamawi Lake and Egg Island, respectively. Brown oval indicates the Athabasca River mouth. Dates of image acquisition are shown.

Athabasca River flow affected the extent of sediment plumes entering Lake Athabasca (Fig 7). Furthermore, lake SSC concentrations were greater in a high flow year (2011) versus a low flow year (2010) (S3 Fig). Differences in SSC were likely responsible for inter-year differences in secchi depth in western Lake Athabasca as water clarity was lower at sites with higher SSC, particularly at sites closer to the mouth of the Athabasca River (S3 Fig). Decreased water clarity was evident in the high flow year.

**Fig 8:**
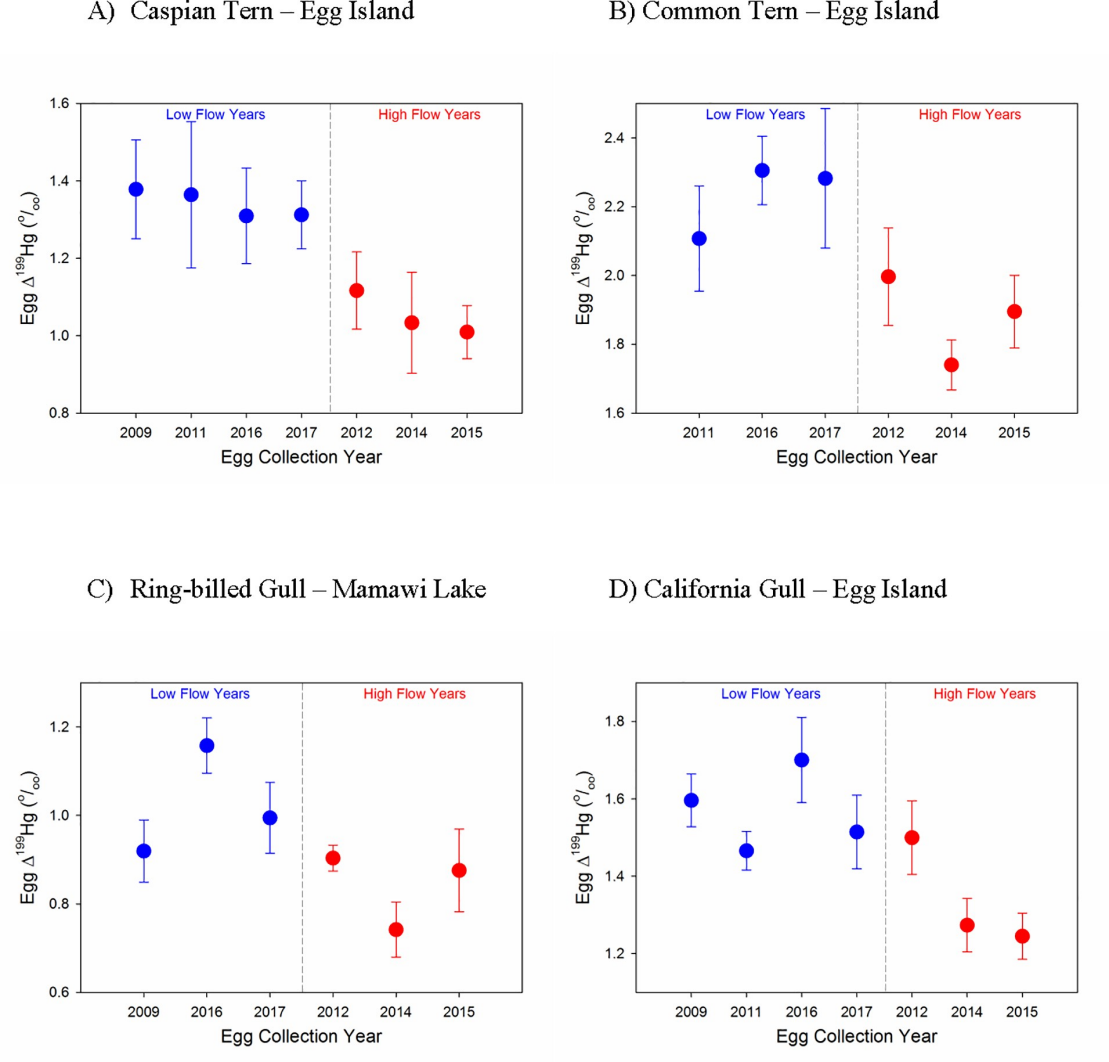
Annual mean (± SE) Δ^199^Hg values (‰) in eggs collected following years of low and high flow in the Athabasca River. Low flow years were categorized as those having a maximal annual flow of less than 1600 m^3^/s. (a) Egg Island Caspian Terns (b) Egg Island Common Terns (c) Mamawi Lake Ring-billed Gulls (d) Egg Island California Gulls.

For each species, differences in egg Δ^199^Hg values were observed following years of low and high Athabasca River flow (Fig 8). Statistical comparison of mean Δ^199^Hg values in eggs collected following low flow years versus high flow years indicated higher Δ^199^Hg values following low flow years in California Gulls (*t* (67) = 3.50, *p* < 0.001), Caspian Terns (*t* (68) = 3.16, *p* = 0.002), Common Terns (*t* (58) = 3.20, *p* = 0.002), Ring-billed Gulls (*t* (55) = 3.18, *p* = 0.002). Similarly, egg Δ^201^Hg values were greater following low flow years than high flow years; California Gulls (*t* (67) = 2.62, *p* = 0.01), Caspian Terns (*t* (68) = 3.16, *p* = 0.002), Common Terns (*t* (58) = 3.50, *p* < 0.001), Ring-billed Gulls (*t* (55) = 5.04, *p* < 0.001) (S6 Table). δ^202^Hg values in eggs did not differ between low and high flow categories for any species (S6 Table).

Δ^199^Hg and Δ^201^Hg values in eggs of all species were correlated (Δ^199^Hg *=* 0.13 + 1.17 * Δ^201^Hg, n = 256, *r* = 0.94, *p* < 0.001). Based upon inter-specific differences between gulls and terns in egg δ^13^C values and δ^202^Hg separate regression analyses were completed for terns and gulls. For gulls, Δ^199^Hg *=* 0.36 + 0.89 * Δ^201^Hg, n = 126, *r* = 0.81, *p* < 0.001, and for terns, Δ^199^Hg *=* 0.06 + 1.27 * Δ^201^Hg, n = 130, *r* = 0.99, *p* < 0.001.

Analysis of epiphytic lichen samples from the Fort McMurray region indicated relatively little spatial variation in Δ^199^Hg (mean across sites = -0.46 ± 0.15‰, individual sample range -0.20 to -0.77‰) or Δ^201^Hg (mean across sites = -0.54 ± 0.17‰, individual sample range -0.18 to -0.86) values (S7 Table). Lichen Δ^199^Hg values were used to estimate baseline MIF of Hg isotopes in atmospherically deposited Hg, an important source of Hg entering the Athabasca River. Lichen Δ^199^Hg values measured here were similar to those in abiotic environmental compartments in the oil sands region, i.e. surface soil (overburden) and road material, bitumen and mined oil sand, processed oil sand, and Athabasca River oil sand [22]. Using egg MIF data for terns from Egg Island and Mamawi Lake, the proportion of MeHg photodegraded was estimated using a modified equation from Bergquist and Blum [30].

$$\ln\left(f\right)=\frac{\left\{1000*\ln\left[0.001(\Delta 199Hgegg-\Delta 199Hglichen)+1\right]\right\}}{S}$$

where *f* = fraction of MeHg photodegraded, Δ199Hgegg = Δ^199^Hg in individual eggs, Δ199Hglichen = average Δ^199^Hg in lichen samples (-0.46), S = -7.82 (from the 10 mg/L dissolved organic carbon (DOC) photochemical demethylation experiment in Bergquist and Blum [30]; June 2015 DOC values in Lake Athabasca ranged from 4–7 mg/L). The amount of MeHg photodegraded was estimated to be 23.0 ± 5.8% (mean ± SD).

## Discussion

In this study, egg THg levels were regulated by species-specific factors, i.e. diet, and by processes associated with the Athabasca River. For the majority of species/site analyses, higher egg THg concentrations were observed in eggs laid following years of high river flow. The threshold effect of river flow on egg THg levels was consistent with Long and Pavelsky’s [12] results demonstrating the influence of river flow on SSC in Lake Athabasca. Enhanced movement of sediment-associated Hg into downstream ecosystems following high river flow may be a critical factor regulating Hg availability to downstream biota. Studies in freshwater rivers have demonstrated the importance of seasonal events on the fate of Hg. In some cases, the annual export of Hg from a watershed can be determined by a single high flow event [14, 44]. Hence, flow was likely of critical importance in moving contaminated sediments to downstream areas with resultant impacts on the bioavailability of Hg to wildlife such as birds.

Two processes may have contributed to higher egg THg levels following years of high river flow. The first stems from the possibility that in high flow years, light attenuation associated with high water SSC may have reduced the amount of photochemical degradation of MeHg. This could have resulted in an increase in the amount of MeHg available for uptake into foodwebs elevating MeHg exposure of laying females with resultant increases in egg THg concentrations. This mechanism cannot likely account for the inter-year differences in egg THg concentrations associated with river flow because only 23% of the MeHg in the tern species was estimated to have been photodegraded. The Athabasca River downstream of Fort McMurray is characterized by high SSC which reduces water transparency and possibly limits the scope for photochemical degradation of MeHg to occur. Despite the fact that photochemical degradation of MeHg was not likely responsible for inter-year fluctuations in egg THg concentrations, inter-year variability in MIF of Hg isotopes was useful in linking river sources of Hg to Hg accumulated in bird eggs. Inter-year differences in egg Δ^199^Hg and Δ^201^Hg values between years of low and high flow provided evidence that riverine processes not only regulated the amount of Hg to which birds were exposed but also the isotopic composition of that Hg.

The slope (1.27) of the Δ^201^Hg/Δ^199^Hg regression for terns was similar to the value associated with photoreduction of MeHg (1.3) indicating that MeHg photoreduction was likely responsible for Δ^199^Hg and Δ^201^Hg values observed in eggs of those species. However, for gulls, a lower slope (0.89) was observed which was more similar to that associated with the photochemical reduction of Hg^2+^ (slope = 1.0 [21]). Tsui et al. [28] reported similar Hg isotope patterns in biota associated with aquatic (slope = 1.20 in benthic invertebrates, slope = 1.24 in trout) versus terrestrial food webs (slope = 1.05). They hypothesized that the mechanisms underlying MIF of Hg differed between aquatic and terrestrial ecosystems. In terrestrial systems, Hg^2+^ may be extensively photoreduced before the non-photoreduced Hg^2+^ undergoes methylation. Hence, differences in Hg sources, pathways of exposure, and environmental Hg processing, could explain the differences that were noted in egg Hg trends across species and sites. For example, isotopic evidence, i.e. egg δ^13^C and δ^202^Hg values, suggested that California Gulls had increased use of terrestrial resources compared to other species, particularly the terns. The less negative egg δ^13^C values in gulls were consistent with what would be expected based upon differences in δ^13^C values of food originating from freshwater and terrestrial ecosystems [45]. Tsui et al. [28] reported higher δ^202^Hg values in organisms associated with terrestrial systems, similar to what was observed here for California Gulls. These results suggested that gulls were incorporating terrestrial foods into their diets in addition to aquatic prey. This is consistent with the known, more omnivorous diet of gulls (see [46] for an example). Despite the fact that all of the bird species studied here are linked to aquatic environments, interspecific differences in dietary pathways of Hg exposure, e.g. obligate aquatic feeders, omnivores, means that different species can provide unique insights into Hg bioaccumulation.

This study provides no direct evidence linking Hg levels in eggs to oil sands sources. However, previous studies have demonstrated that oil sands developments are a source of Hg to the local environment [2, 3]. For example, Hg in snow was 5.6 times greater within 50 km of oil sands processing facilities than outside that area [2]. Hg levels in water sampled in summer were three times greater downstream of areas disturbed by oil sands development than at upstream locations situated in the mineable oil sands region [2]. Water Hg concentrations further downstream in the Athabasca River and PAD were two times greater than these upstream concentrations [2]. Elevated levels of Hg in abiotic environmental matrices are also reflected in biota. For example, THg concentrations in prey fish sampled from the Athabasca River in the surface mineable oil sands region were five times greater than fish collected from the Athabasca River upstream of the oil sands [6]. THg levels in prey fish collected in the PAD and Lake Athabasca were two to four times greater than fish from the upstream Athabasca River site [6]. THg levels in eggs of California Gulls and Herring Gulls (*Larus argentatus*) were two and three times greater, respectively, at Egg Island (Lake Athabasca) than at Namur Lake, an inland lake isolated from the Athabasca River but in close proximity (~60 km west) to open-pit oil sands mines [6]. Taken together, these results indicate that the oil sands region is a source of Hg to the environment and that biota inhabiting waters in or downstream of the oil sands have higher Hg levels. To understand this further, an integrated research program involving Hg measurements in air, water, land, and biota is necessary to assess the relative importance of oil sands sources as a contributor to the overall Hg budget of the Athabasca River and downstream ecosystems. With respect to bird eggs, we can begin to predict expected Hg levels based upon river flow. For example, in 2017, mean June river flow was high (~1580 m^3^/s), hence egg THg levels in 2018 are also predicted to be high.

Until now, uncertainty surrounded the degree to which Hg may be transported by the Athabasca River to ecosystems far downstream. Following spring melt of the snowpack in the Athabasca River watershed, it is likely that some of the snow-associated mercury finds its way into the river. This is particularly true in the spring when frozen soils may limit infiltration of runoff into soil leading to efficient Hg export to the Athabasca River from overland sources [47, 48]. Furthermore, during high flow years, Athabasca River sediments are mobilized and transported to downstream environments such as the PAD and western Lake Athabasca [12]. River systems can convey 90% of their total heavy metal load via sediment transport [13, 44, 48]. Hence, it is highly likely that Athabasca River sediments are transporting Hg to areas far downstream. This hypothesis was supported by Long and Pavelsky’s [38] data and by satellite images showing inter-year differences in sediment plumes into Lake Athabasca. Aquatic bird egg THg concentrations and Hg isotope data indicate that this Hg is being incorporated into downstream food webs.

The current study highlights the degree to which local inputs of Hg to the river via atmospheric releases, mobilization associated with land disturbance, or dust/leakage from roads, tailings ponds (but see [49]), and other sources will not remain confined to the local receiving environment. Hg from these sources will be transported long distances via the river to sensitive downstream ecosystems, e.g. PAD, WBNP, that are recognized for their unique, world-class ecological characteristics. New surface-mine oil sands projects are being proposed that will bring development much closer (~30 km) to the southern boundary of the PAD/WBNP. This will likely increase Hg inputs into the local environment through Hg mobilization stemming from further land development and atmospheric Hg releases. The zone of atmospheric deposition may encompass southern parts of the PAD/WBNP based upon the size of depositional zones previously characterized for oil sands operations [2, 3]. Hence, further oil sands development along with expansion of other human activities that disturb the landscape, e.g. road-building, forestry, may result in increased delivery of Hg to downstream/downwind areas. The impacts of multiple stressors on WBNP have resulted in it being investigated as an UNESCO World Heritage Site in Danger. Cumulative impact assessment of existing and proposed industrial projects needs to consider potential Hg impacts on wildlife and humans inhabiting this globally-recognized area of ecological and cultural importance.

## Supporting information

S1 Fig Surface-mineable oil sands production (synthetic oil and bitumen) in millions of barrels per day, 1967–2017. Data are from CAPP [1]. The dashed line indicates the beginning of the period during which egg samples were collected to assess temporal trends in mercury levels.
(TIF)

S2 Fig Mean June flow (m^3^/s) of the Athabasca River north of Fort McMurray (station 07DA001) from 1958–2017. The dashed line indicates the beginning of the period during which the influence of river flow on egg THg levels was investigated. Data are from Canadian Hydrographic Service (https://wateroffice.ec.gc.ca/mainmenu/historical_data_index_e.html).
(TIF)

S3 Fig Suspended sediment concentrations (SSC) (mg/L) and secchi disc depth (cm) along a transect from a point (58.6724 N, -110.8477 W) at the mouth of the Athabasca River. Data for a low flow (2010) and a high flow year (2011) are shown. Data are from Long and Pavelsky [38].
(TIF)

S1 Table Total mercury (THg) and isotope data measured in aquatic bird eggs, 2009–2017. (XLSX)

S2 Table Annual egg THg concentrations by species and site and various possible influencing factors. (XLSX)

S3 Table Results of information-theoretic analysis comparing models using Akaike’s Information Criterion (AIC) adjusted for small sample sizes (AICc). Model weights (w) are also shown.
(XLSX)

S4 Table Annual mean (± 1 SD) δ^13^C values (‰) in eggs of California Gulls (CAGU), Caspian Terns (CATE), Common Terns (COTE), and Ring-billed Gulls (RBGU) collected from Egg Island and Mamawi Lake. δ^13^C values were adjusted for lipid content. At each site, inter-year differences in species-specific δ^13^C values were evaluated using ANOVA/Tukey’s HSD or Kruskal-Wallis/Dunn’s tests. Superscript letters indicate statistically significant differences (*p* < 0.05) between years. Means with the same letter are not different. n is the number of samples analyzed for each species at each site.
(DOCX)

S5 Table Annual mean (± 1 SD) δ^15^N values (‰) in eggs of California Gulls (CAGU), Caspian Terns (CATE), Common Terns (COTE), and Ring-billed Gulls (RBGU) collected from Egg Island and Mamawi Lake. At each site, inter-year differences in species-specific δ^15^N values were evaluated using ANOVA or Kruskal-Wallis/Dunn’s tests. Superscript letters indicate statistically significant differences (*p* < 0.05) between years. Means with the same letter are not different. n is the number of samples analyzed for each species at each site.
(DOCX)

S6 Table Annual mean (‰) mass dependent fractionation (MDF, δ^202^Hg) and mass-independent fractionation (MIF, Δ^201^Hg) of Hg isotopes in aquatic bird eggs. California Gulls (CAGU), Caspian Terns (CATE), Common Terns (COTE), and Ring-billed Gulls (RBGU) collected from Egg Island and Mamawi Lake. Athabasca River flow in the year preceding egg collections was categorized as low or high (≥ 1600 m^3^/s in June) for each year. MDF and MIF values were compared between flow categories for each species. * indicates statistically significant differences (t-test, *p* < 0.05) between flow categories. n is the number of egg samples included in each category.
(DOCX)

S7 Table Mercury isotopes (‰) in lichen samples from northern Alberta. (XLSX)

## References

[1] CAPP (Canadian Association of Petroleum Producers) (2018) Statistical Handbook. https://www.capp.ca/publications-and-statistics/statistics. Accessed Feb 5, 2019.

[2] Kelly EN, Schindler DW, Hodson PV, Short JW, Radmanovich R, Nielsen CC (2010) Oil sands development contributes elements toxic at low concentrations to the Athabasca River and its tributaries. Proc Natl Acad Sci USA 107: 16178−16183. 10.1073/pnas.1008754107 20805486 PMC2941314

[3] Kirk JL, Muir DCG, Gleason A, Wang XW, Lawson G, Frank RA, et al (2014) Atmospheric deposition of mercury and methylmercury to landscapes and waterbodies of the Athabasca Oil Sands region. Environ Sci Technol 48: 7374–7383. 10.1021/es500986r 24873895

[4] Raikow DF, Walters DM, Fritz KM, Mills MA (2011) The distance that contaminated aquatic subsidies extend into lake riparian zones. Ecol Appl 21: 983–990. 21639060 10.1890/09-1504.1

[5] Bartons M, Gratton C, Spiesman BJ, Vander Zanden MJ (2015) Taking the trophic bypass: aquatic-terrestrial linkage reduces methylmercury in a terrestrial food web. Ecol Appl 25: 151–159. 26255364 10.1890/14-0038.1

[6] Dolgova S, Popp BN, Courtoreille K, Espie RHM, Maclean B, McMaster M, et al (2018) Spatial trends in a biomagnifying contaminant: application of amino acid compound specific stable nitrogen isotope analysis to the interpretation of bird mercury levels. Environ Toxicol Chem 37: 1466−1475. 10.1002/etc.4113 29446488

[7] Hebert CE, Popp BN (2018) Temporal trends in a biomagnifying contaminant: Application of amino acid compound specific stable nitrogen isotope analysis to the interpretation of bird mercury levels. Environ Toxicol Chem 37: 1458–1465. 10.1002/etc.4092 29341238

[8] Biswas A, Blum JD, Klaue B, Keeler GJ (2007) Release of mercury from Rocky Mountain forest fires. Global Biogeochem Cycles. 10.1029/2006GB002696

[9] Wiedinmyer C, Friedli H (2007) Mercury emission estimates from fires: an initial inventory for the United States. Environ Sci Technol 41: 8092–8098. 18186342 10.1021/es071289o

[10] National Forestry Database (2018). Annual forest area burned for Alberta. National Resources Canada, Ottawa, ON, Canada http://nfdp.ccfm.org/en/data/fires.php. Accessed Feb 5, 2019.

[11] Hebert CE, Weseloh DVC, MacMillan S, Campbell D, Nordstrom W (2011) Metals and PAHs in colonial waterbird eggs from Lake Athabasca and the Peace-Athabasca Delta, Canada. Environ Toxicol Chem 30: 1178–1183. 10.1002/etc.489 21312251

[12] Long CM, Pavelsky TM (2013) Remote sensing of suspended sediment concentration and hydrologic connectivity in a complex wetland environment. Remote Sens Environ 129: 197–209.

[13] Carroll RWH, Warwick JJ, Heim KJ, Bonzongo JC, Miller JR, Lyons WB (2000) Simulation of mercury transport and fate in the Carson River, Nevada. Ecol Model 125: 255–278.

[14] Saniewska D, Be1dowska M, Be1dowski J, Jedruch A, Saniewski M, Falkowska L (2014) Mercury loads into the sea associated with extreme flood. Environ Pollut 191: 93–100. 10.1016/j.envpol.2014.04.003 24816201

[15] Hebert CE, Norstrom RJ, Weseloh DV (1999) A quarter century of environmental surveillance: the Canadian Wildlife Service’s Great Lakes Herring Gull Monitoring Program. Environ Rev 7: 147–166.

[16] Hobson KA, Hughes KD, Ewins PJ (1997) Using stable isotope analysis to identify endogenous and exogenous sources of nutrients in eggs of migratory birds: Applications to Great Lakes contaminants research. Auk 114: 467–478.

[17] Ramsar (2016) The Ramsar Convention Manual: a Guide to the Convention on Wetlands, 4th ed Ramsar Convention Secretariat, Gland, Switzerland.

[18] Kuyt E (1993) Whooping crane, *Grus americana*, home range and breeding range expansion in Wood Buffalo National Park, 1970−1991. Can Field Nat 107: 1−12.

[19] Hebert CE, Nordstrom W, Shutt JL (2010) Colonial waterbirds nesting on Egg Island, Lake Athabasca. Can Field Nat 124: 49−53.

[20] Chan L, Receveur O, Batal M, David W, Schwartz H, Ing A, et al (2016) First Nations Food, Nutrition and Environment Study (FNFNES): Results from Alberta 2013. University of Ottawa, Ottawa, ON, pp. 155.

[21] Bargagli R, Barghigiani C (1991) Lichen biomonitoring of mercury emission and deposition in mining, geothermal and volcanic areas of Italy. Environ Monit Assess 16: 265−275. 10.1007/BF00397614 24241939

[22] Blum J, Johnson M, Gleason J, Demers J, Landis M, Krupa S (2012) Mercury concentration and isotopic composition of epiphytic tree lichens in the Alberta oil sands region In: Percy KE, ed. Alberta Oil Sands: Energy, Industry, and The Environment. Elsevier Ltd, Oxford, UK pp 373–390.

[23] Ackerman JT, Herzog MP, Schwarzbach SE (2013) Methylmercury is the dominant form of mercury in bird eggs: A synthesis. Environ Sci Technol 47: 2052−2060. 10.1021/es304385y 23331121

[24] Kelly JF (2000) Stable isotopes of carbon and nitrogen in the study of avian and mammalian trophic ecology. Can J Zool 78: 1–27.

[25] Elliott KH, Davis M, Elliott JE (2014) Equations for lipid normalization of carbon stable isotope ratios in aquatic bird eggs. PLOS ONE 9: e83597 10.1371/journal.pone.0083597 24465384 PMC3898914

[26] Georg RB, Newman K (2015) The effect of hydride formation on instrumental mass discrimination in MC-ICP-MS: a case study of mercury (Hg) and thallium (Tl) isotopes. J Anal At Spectrom 30: 1935–1944.

[27] Blum JD, Sherman SL, Johnson ML (2014) Mercury isotopes in earth and environmental sciences. Annu Rev Earth Planet Sci 42: 249–69.

[28] Tsui MTK, Blum JD, Finlay JC, Balogh SJ, Nollet YH, Palen WJ, et al (2014) Variation in terrestrial and aquatic sources of methylmercury in stream predators as revealed by stable mercury isotopes. Environ Sci Technol 48: 10128–10135. 10.1021/es500517s 25105808

[29] Kwon SY, Blum JD, Carvan MJ, Basu N, Head JA, Madenjian CP, et al (2012) Absence of fractionation of mercury isotopes during trophic transfer of methylmercury to freshwater fish in captivity. Environ Sci Technol 46: 7527–7534. 10.1021/es300794q 22681311 PMC4347840

[30] Bergquist BA, Blum JD (2007) Mass-dependent and–independent fractionation of Hg isotopes by photoreduction in aquatic systems. Science 318: 417−420. 10.1126/science.1148050 17872409

[31] Tsui MTK, Blum JD, Finlay JC, Balogh SJ, Kwon SY, Nollet YH (2013) Photodegradation of methylmercury in stream ecosystems. Limnol Oceanogr 58: 13–22.

[32] Point D, Sonke JE, Day RD, Roseneau DG, Hobson KA, Vander Pol SS, et al (2011) Methylmercury photodegradation influenced by sea-ice cover in Arctic marine ecosystems. Nature Geoscience 4: 188–194.

[33] Day RD, Roseneau DG, Berail S, Hobson KA, Donard OFX, Vander Pol SS, et al (2012) Mercury stable isotopes in seabird eggs reflect a gradient from terrestrial geogenic to oceanic mercury reservoirs. Environ Sci Technol 46: 5327−5335. 10.1021/es2047156 22519440

[34] Sherman LS, Blum JD (2013) Mercury stable isotopes in sediments and largemouth bass from Florida lakes, USA. Sci Total Environ 448: 163–175. 10.1016/j.scitotenv.2012.09.038 23062970

[35] Caldwell CA, Canavaan CM, Bloom NS (2000) Potential effects of a forest fire and storm flow on total mercury and methylmercury in sediments of an arid-lands reservoir. Sci Total Environ 260: 125–133. 11032121 10.1016/s0048-9697(00)00554-4

[36] Harris RC, Rudd JWM, Amyot M, Babiarz CL, Beaty KG, Blanchfield PJ, et al (2007) Whole-ecosystem study shows rapid fish-mercury response to changes in mercury deposition. Proc Natl Acad Sci USA 104: 16586–16591. 10.1073/pnas.0704186104 17901207 PMC2034227

[37] USGS (United States Geological Survey) (2018) Landsat Missions. https://landsat.usgs.gov/. Accessed Feb 5, 2019.

[38] Long CM, Pavelsky TM (2012) Water Quality and Spectral Reflectance, Peace-Athabasca Delta, Canada, 2010–2011. Data set. 2012. Available online [http://daac.ornl.gov] from Oak Ridge National Laboratory Distributed Active Archive Center, Oak Ridge, Tennessee, U.S.A. 10.3334/ORNLDAAC/1133. Accessed Feb 5, 2019.

[39] Burnham KP, Anderson DR (2002) Model Selection and Multimodel Inference: A Practical Information-Theoretic Approach. 2nd ed Springer-Verlag, New York. Pp. 488.

[40] StatSoft (2013). Statistica (Data Analysis Software System), Ver 12. Tulsa, OK, USA.

[41] Schindler DW (2001) The cumulative effects of climate warming and other human stresses on Canadian freshwaters in the new millennium. Can J Fish Aquat Sci 58: 18–29.

[42] Schindler DW, Donahue WF (2006). An impending water crisis in Canada's western prairie provinces. Proc Natl Acad Sci USA 103: 7210–7216. 10.1073/pnas.0601568103 16606829 PMC1564278

[43] Dibikea Y, Eumb H, Prowse T (2018) Modelling the Athabasca watershed snow response to a changing climate. J Hydrol: Regional Studies 15: 134−148.

[44] Babiarz CL, Hurley JP, Benoit JM, Shafer MM, Andren AW, Webb DA (1998) Seasonal influences on partitioning and transport of total and methyl–mercury in rivers from contrasting watersheds. Biogeochem 41: 237–257.

[45] Hebert CE, Shutt JL, Hobson KA, Weseloh DV (1999) Spatial and temporal differences in the diet of Great Lakes herring gulls (*Larus argentatus*): Evidence from stable isotope analysis. Can J Fish Aquatic Sci 56: 323–338.

[46] Nisbet IC, Weseloh DV, Hebert CE, Mallory ML, Poole AF, Ellis JC, et al (2017) Herring Gull (*Larus argentatus*) In: Rodewald PG, ed. The Birds of North America. Cornell Lab of Ornithology, Ithaca, NY Retrieved from the Birds of North America: https://birdsna.org/Species-Account/bna/species/hergul. Accessed Feb 5, 2019.

[47] Hurley JP, Shafer MM, Cowell SE, Overdier JT, Hughes PE, Armstrong DE (1996) Trace metal assessment of Lake Michigan tributaries using low-level techniques. Environ Sci Technol 30: 2093–2098.

[48] Balogh SJ, Meyer ML, Johnson DK (1997) Mercury and suspended sediment loadings in the lower Minnesota River. Environ Sci Technol 31: 198–202.

[49] Willis CE, St Louis VL, Kirk JL, St Pierre KA, Dodge C (2018) Tailings ponds of the Athabasca Oil Sands Region, Alberta, Canada, are likely not significant sources of total mercury and methylmercury to nearby ground and surface waters. Sci Total Environ 647: 1604–1610. 10.1016/j.scitotenv.2018.08.083 30180364

