# Supplementary figures and images for: The river runs through it: The Athabasca River delivers mercury to aquatic birds breeding far downstream
Source: PLoS One. 2019 Apr 9;14(4):e0206192. doi: 10.1371/journal.pone.0206192 (PMC6456287; doi:10.1371/journal.pone.0206192)

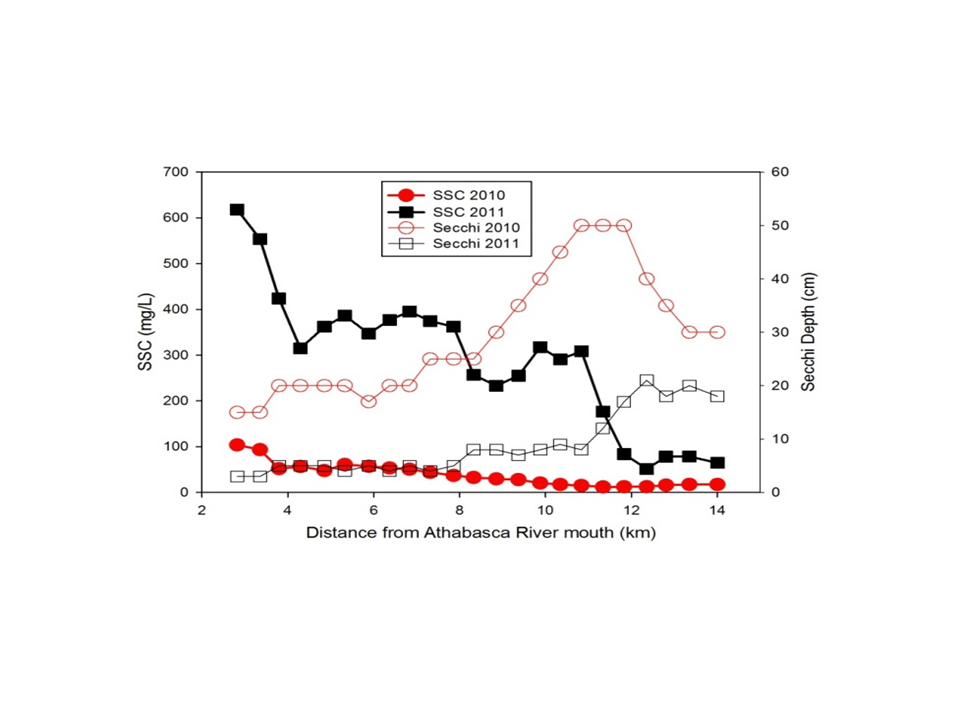

Supplement: S3 Fig — Data for a low flow (2010) and a high flow year (2011) are shown. Data are from Long and Pavelsky [38]. (TIF) [file pone.0206192.s003.tif]
