# Supplementary material for: The river runs through it: The Athabasca River delivers mercury to aquatic birds breeding far downstream
Source: PLoS One. 2019 Apr 9;14(4):e0206192. doi: 10.1371/journal.pone.0206192 (PMC6456287; doi:10.1371/journal.pone.0206192)
Supplement: S4 Table — δ13C values were adjusted for lipid content. At each site, inter-year differences in species-specific δ13C values were evaluated using ANOVA/Tukey’s HSD or Kruskal-Wallis/Dunn’s tests. Superscript letters indicate statistically significant differences (p < 0.05) between years. Means with the same letter are not different. n is the number of samples analyzed for each species at each site. (DOCX) [file pone.0206192.s007.docx]

**Table S4.** Annual mean (± 1 SD) δ^13^C values (‰) in eggs of California Gulls (CAGU), Caspian Terns (CATE), Common Terns (COTE), and Ring-billed Gulls (RBGU) collected from Egg Island and Mamawi Lake. δ^13^C values were adjusted for lipid content. At each site, inter-year differences in species-specific δ^13^C values were evaluated using ANOVA/Tukey’s HSD or Kruskal-Wallis/Dunn’s tests. Superscript letters indicate statistically significant differences (*p* < 0.05) between years. Means with the same letter are not different. n is the number of samples analyzed for each species at each site.

|  | Egg Island, Lake Athabasca | | | | | | Mamawi Lake, Peace-Athabasca Delta | | | |
| --- | --- | --- | --- | --- | --- | --- | --- | --- | --- | --- |
|  | CAGU | | CATE | | COTE | | RBGU | | COTE | |
| Year | Mean | SD | Mean | SD | Mean | SD | Mean | SD | Mean | SD |
| 2009 | -23.88 | 1.36 | -25.66^abc^ | 2.88 |  |  | -25.46^ab^ | 1.33 | -26.25^abc^ | 1.41 |
| 2011 | -23.58 | 0.94 | -24.39^a^ | 2.21 | -24.72^a^ | 2.27 |  |  |  |  |
| 2012 | -22.83 | 0.73 | -25.41^ab^ | 0.98 | -26.50^ab^ | 1.00 | -25.53^ab^ | 0.49 | -25.35^ab^ | 0.37 |
| 2013 | -23.58 | 0.89 | -25.08^a^ | 2.08 | -26.12^ab^ | 0.26 | -24.14^a^ | 1.13 | -24.56^a^ | 0.95 |
| 2014 | -23.94 | 0.84 | -27.98^c^ | 1.32 | -26.65^abc^ | 1.14 | -27.01^b^ | 2.14 |  |  |
| 2015 | -23.98 | 1.00 | -27.56^bc^ | 1.71 | -27.87^c^ | 0.55 | -27.01^b^ | 2.34 | -27.22^c^ | 0.65 |
| 2016 | -24.09 | 0.84 | -25.78^abc^ | 0.86 | -27.14^bc^ | 0.63 | -25.28^ab^ | 1.27 | -26.82^bc^ | 0.59 |
| 2017 | -23.40 | 1.31 | -24.48^a^ | 1.24 | -26.72^abc^ | 0.63 | -25.64^ab^ | 0.67 | -26.57^abc^ | 0.44 |
| Mean | -23.67 | 1.04 | -25.79 | 2.11 | -26.51 | 1.43 | -25.71 | 1.58 | -26.09 | 1.24 |
| N | 81 |  | 80 |  | 70 |  | 83 |  | 55 |  |
