# Supplementary material for: The river runs through it: The Athabasca River delivers mercury to aquatic birds breeding far downstream
Source: PLoS One. 2019 Apr 9;14(4):e0206192. doi: 10.1371/journal.pone.0206192 (PMC6456287; doi:10.1371/journal.pone.0206192)
Supplement: S5 Table — At each site, inter-year differences in species-specific δ15N values were evaluated using ANOVA or Kruskal-Wallis/Dunn’s tests. Superscript letters indicate statistically significant differences (p < 0.05) between years. Means with the same letter are not different. n is the number of samples analyzed for each species at each site. (DOCX) [file pone.0206192.s008.docx]

**Table S5.** Annual mean (± 1 SD) δ^15^N values (‰) in eggs of California Gulls (CAGU), Caspian Terns (CATE), Common Terns (COTE), and Ring-billed Gulls (RBGU) collected from Egg Island and Mamawi Lake. At each site, inter-year differences in species-specific δ^15^N values were evaluated using ANOVA or Kruskal-Wallis/Dunn’s tests. Superscript letters indicate statistically significant differences (*p* < 0.05) between years. Means with the same letter are not different. n is the number of samples analyzed for each species at each site.

|  | Egg Island, Lake Athabasca | | | | | | Mamawi Lake, Peace-Athabasca Delta | | | |
| --- | --- | --- | --- | --- | --- | --- | --- | --- | --- | --- |
|  | CAGU | | CATE | | COTE | | RBGU | | COTE | |
| Year | Mean | SD | Mean | SD | Mean | SD | Mean | SD | Mean | SD |
| 2009 | 9.44 | 1.11 | 10.76 | 0.80 |  |  | 8.64 | 2.02 | 11.41^ab^ | 1.75 |
| 2011 | 9.68 | 0.93 | 11.07 | 0.86 | 10.34 | 0.69 |  |  |  |  |
| 2012 | 9.61 | 1.05 | 11.02 | 0.53 | 10.41 | 0.70 | 9.06 | 1.07 | 10.69^ab^ | 0.24 |
| 2013 | 10.69 | 0.71 | 11.60 | 1.01 | 10.51 | 0.29 | 9.64 | 1.04 | 10.41^b^ | 0.29 |
| 2014 | 9.71 | 0.76 | 11.29 | 0.74 | 10.48 | 0.57 | 8.83 | 1.27 |  |  |
| 2015 | 10.19 | 0.79 | 11.33 | 0.44 | 10.28 | 0.36 | 9.48 | 0.77 | 11.35^a^ | 0.35 |
| 2016 | 9.77 | 0.64 | 11.28 | 0.52 | 10.12 | 0.23 | 8.61 | 0.69 | 10.45^b^ | 0.28 |
| 2017 | 9.89 | 0.78 | 11.44 | 1.19 | 10.11 | 0.21 | 8.47 | 1.00 | 10.43^b^ | 0.28 |
| Mean | 9.87 | 0.90 | 11.22 | 0.80 | 10.32 | 0.48 | 8.89 | 1.20 | 10.82 | 0.88 |
| n | 81 |  | 80 |  | 70 |  | 83 |  | 55 |  |

Egg stable nitrogen isotope values (δ^15^N) were different among all species comparisons (Kruskal-Wallis *H*(3,369) = 171.85, *p* < 0.001, Dunn’s test, Caspian Tern > Common Tern > California Gull > Ring-billed Gull). δ^15^N values within most species and sites showed no differences across years (Egg Island: California Gulls ANOVA *F*(7,73) = 2.12, *p* = 0.051; Caspian Terns ANOVA *F*(7,72) = 1.09, *p* = 0.38; Common Terns Kruskal-Wallis *H*(6,70) = 9.37, *p* = 0.15; Mamawi Lake: Ring-billed Gulls Kruskal-Wallis *H*(6,83) = 12.86, *p* = 0.05, Dunn’s post-hoc test no differences). Only Mamawi Lake Common Terns showed significant inter-year differences in egg δ^15^N values (Kruskal-Wallis *H*(5,55) = 20.76, *p* = 0.01, Dunn’s test).
