# Supplementary material for: The river runs through it: The Athabasca River delivers mercury to aquatic birds breeding far downstream
Source: PLoS One. 2019 Apr 9;14(4):e0206192. doi: 10.1371/journal.pone.0206192 (PMC6456287; doi:10.1371/journal.pone.0206192)
Supplement: S6 Table — California Gulls (CAGU), Caspian Terns (CATE), Common Terns (COTE), and Ring-billed Gulls (RBGU) collected from Egg Island and Mamawi Lake. Athabasca River flow in the year preceding egg collections was categorized as low or high (≥ 1600 m3/s in June) for each year. MDF and MIF values were compared between flow categories for each species. * indicates statistically significant differences (t-test, p < 0.05) between flow categories. n is the number of egg samples included in each category. (DOCX) [file pone.0206192.s009.docx]

**Table S6.** Annual mean (‰) mass dependent fractionation (MDF, δ^202^Hg) and mass-independent fractionation (MIF, Δ^201^Hg) of Hg isotopes in aquatic bird eggs. California Gulls (CAGU), Caspian Terns (CATE), Common Terns (COTE), and Ring-billed Gulls (RBGU) collected from Egg Island and Mamawi Lake. Athabasca River flow in the year preceding egg collections was categorized as low or high (≥ 1600 m^3^/s in June) for each year. MDF and MIF values were compared between flow categories for each species. * indicates statistically significant differences (t-test, *p* < 0.05) between flow categories. n is the number of egg samples included in each category.

|  |  | Egg Island, Lake Athabasca | | | | | | Mamawi Lake, Peace-Athabasca Delta | |
| --- | --- | --- | --- | --- | --- | --- | --- | --- | --- |
| Collection Year |  | CAGU | | CATE | | COTE | | RBGU | |
|  | Flow | δ^202^Hg | Δ^201^Hg | δ^202^Hg | Δ^201^Hg | δ^202^Hg | Δ^201^Hg | δ^202^Hg | Δ^201^Hg |
| 2009 | Low | 0.78 | 1.27 | -0.49 | 1.04 |  |  | -0.43 | 0.77 |
| 2011 | Low | 0.60 | 1.20 | -0.21 | 1.02 | 0.10 | 1.64 |  |  |
| 2012 | High | 0.58 | 1.23 | -0.32 | 0.83 | 0.03 | 1.54 | 0.01 | 0.66 |
| 2014 | High | 0.91 | 1.00 | -0.51 | 0.76 | -0.48 | 1.28 | -0.24 | 0.48 |
| 2015 | High | 0.89 | 1.01 | -0.46 | 0.76 | -0.13 | 1.43 | -0.28 | 0.59 |
| 2016 | Low | 1.16 | 1.22 | -0.24 | 0.97 | -0.07 | 1.76 | -0.13 | 0.98 |
| 2017 | Low | 1.11 | 1.27 | -0.23 | 1.00 | 0.01 | 1.73 | -0.03 | 0.87 |
| Mean | Low | 0.90 | 1.24* | -0.29 | 1.01* | 0.01 | 1.71* | -0.20 | 0.87* |
|  | n | 42 |  | 40 |  | 31 |  | 29 |  |
| Mean | High | 0.79 | 1.08 | -0.43 | 0.78 | -0.20 | 1.41 | -0.17 | 0.57 |
|  | n | 27 |  | 30 |  | 29 |  | 28 |  |
